# Identification of a Nonribosomal Peptide Analog With Activity Against Multiple Gram‐Positive Bacteria via a Synthetic Bioinformatic Natural Product Discovery Approach

**DOI:** 10.1002/advs.76103

**Published:** 2026-06-11

**Authors:** Keyi Chen, Jiayi Liang, Yujia Wu, Jianan Xu, Yi Liu, Wenguang Wang, Xinhang Jiang, Benjie Gao, Yueyue Wang, Hui Jiang

**Affiliations:** ^1^ Institute of Biochemistry College of Life Sciences Zhejiang University Hangzhou China; ^2^ Liangzhu Laboratory Zhejiang University Medical Center Hangzhou China; ^3^ ZJU‐UoE Joint Research Centre for Engineering Biology International Campus of Zhejiang University Zhejiang University Haining China; ^4^ Zhejiang University‐Lishui Joint Innovation Center for Life and Health & Lishui Lvgu Institute for Life and Health Lishui China

**Keywords:** antibiotics, nonribosomal peptides, structure‐activity relationships, syn‐BNPs

## Abstract

Nonribosomal peptide (NRP) antibiotics exhibit potent biological activities and are broadly used in clinical therapy. Because most microorganisms are difficult to culture and many antibiotic biosynthetic genes are silent, traditional activity tracking approaches face major limitations in the discovery of novel NRPs. Here, based on a synthetic bioinformatic natural product (syn‐BNP) discovery approach that integrates bioinformatics and chemical synthesis, a novel nonribosomal peptide synthetase (NRPS) gene cluster from the genome of *Rhodococcus erythropolis* D‐1 was mined. A putative NRP scaffold synthesized by the NRPS encoded by this cluster was predicted. Through chemical synthesis and four rounds of structure‐activity relationship (SAR) studies, 37 NRP analogs were ultimately generated. Among these analogs, ZURJC28 shows activity against multiple Gram‐positive bacteria, including two drug‐resistant strains. Mechanistic studies and metabolomics analyses revealed that ZURJC28 exerts membrane‐disruptive activity associated with interaction with phosphatidylglycerol (PG)‐enriched Gram‐positive membranes, leading to membrane damage and widespread metabolic dysregulation. ZURJC28 also shows low cytotoxicity and low hemolytic activity, suggesting its preliminary in vitro safety profile.

## Graphical abstract

The genome of *Rhodococcus erythropolis* D‐1 was analyzed by bioinformatic tools to mine a novel nonribosomal peptide synthetase (NRPS) gene cluster. A nonribosomal peptide analog ZURJC5 associated with the NRPS was chemically synthesized. Through structure‐activity relationship studies, ZURJC28 was ultimately obtained and showed antibacterial activity. Mechanistic studies of ZURJC28 revealed that it exerts membrane‐disruptive activity associated with interaction with phosphatidylglycerol (PG)‐enriched Gram‐positive membranes.

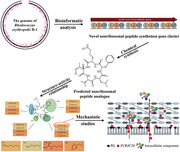

## 1 Introduction

Multidrug‐resistant bacteria have become an inescapable challenge in modern medicine, and the evolution of resistant strains far exceeds the discovery of new antibiotics by traditional strategies [1, 2]. Hence, establishing a rapid and efficient discovery strategy for new antibiotics is of great significance to global public health [3]. The classical method for discovering novel antibiotics is generally based on activity tracking, which enables the isolation of active antibiotics from microbial fermentation products [4, 5]. Nevertheless, this strategy is limited by the challenges associated with difficulty of culturing most microbes [6] and silent antibiotic gene clusters [7, 8]. The novelty of isolated antibiotics is also difficult to determine at early stages in the traditional strategy, leading researchers to explore more efficient strategies [9].

Nonribosomal peptides (NRPs) are an important class of natural antibiotics [10, 11], and many NRPs discovered from microbes exhibit significant pharmacological activities, such as penicillin [12], bleomycin [13], and cyclosporin A [14]. NRPs are synthesized by nonribosomal peptide synthetases (NRPSs). NRPS consists of many modules, and each module contains several domains, among which the adenylation (A) domain specifically activates amino acid substrates and collaborates with other domains to synthesize NRPs [15]. Thus, bioinformatic analysis of NRPS biosynthetic gene clusters (BGCs) enables the prediction of the NRP structural skeleton, guiding subsequent chemical synthesis of these putative peptides. This strategy is known as the synthetic bioinformatic natural products (syn‐BNPs) discovery approach [16]. For instance, Vila‐Farres and colleagues predicted 280 NRPS BGCs using a syn‐BNP discovery approach and obtained 288 corresponding peptides, among which Syn‐BNP 1 exhibited broad‐spectrum antibacterial activity [17].

Since current bioinformatics tools remain limited, the syn‐BNP discovery approach has difficulty predicting peptide cyclization patterns and N‐terminal fatty acid modifications, resulting in an extremely large number of analogs need to be synthesized [18]. Notably, whether starting from natural products or predicted natural product analogs, both approaches require constructing and screening a large library of natural product analogs for subsequent screening, leading to the time required for the two overall drug development processes is roughly the same.

Actinomycetes are a major source of NRPS BGCs [19, 20]. Currently, research on NRPs in *Streptomyces* is relatively comprehensive. *Rhodococcus* is a rare actinomycete with diverse metabolic capabilities and strong pollutant treatment abilities. Their genomes are rich in NRPS BGCs, conferring considerable potential for the discovery of novel natural antibiotics [21].

This study started from eight NRP analogs associated with a NRPS BGC in the genome of *Rhodococcus erythropolis* D‐1 [22], followed by systematic synthesis of 29 additional analogs through four rounds of structure‐activity relationship (SAR) studies. Ultimately, ZURJC28 showed the lowest MIC of 4 µg/mL against *Staphylococcus epidermidis* CGMCC 1.16091. We further studied antibacterial mechanism and analyzed metabolome on ZURJC28. Overall, by incorporating SAR studies with the syn‐BNP discovery approach, we explored ZURJC28 with antibacterial activities rapidly and economically.

## 2 Results

### 2.1 Characterization of Antibacterial Compounds From *R. erythropolis* D‐1 via the Cross‐Streak Method

**FIGURE 1:**
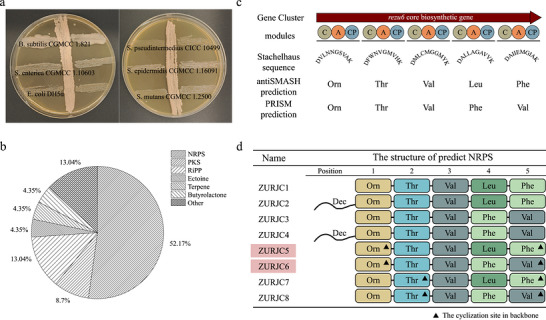
Antibacterial activity of *R. erythropolis* D‐1 culture and bioinformatic analysis of the *R. erythropolis* D‐1 genome. (a) The cross‐streak method of *R. erythropolis* D‐1 culture. The results showed no inhibition zones toward several model bacteria. (b) Bioinformatic analysis of the *R. erythropolis* D‐1 genome. The ratio of NRPS BGCs to all BGCs is the highest, accounting for 52.17%. (c) The core biosynthetic gene of the *rezu6* NRPS BGC. The *rezu6* NRPS is predicted to have five modules, each of which contain a C (condensation)‐ and A (adenylation)‐ and CP (peptidyl carrier protein)‐domain. The substrate specificities of the A domains were predicted on the basis of the Stachelhaus sequence by antiSMASH and PRISM. (d) The eight original NRP analogs based on the predicted skeleton biosynthesized from the *rezu6* NRPS. The triangle represents the cyclization sites in the backbone. The red square represents the two active NRP analogs. Different color groups represent different amino acid properties: yellow for basic amino acids, blue for neutral hydrophilic amino acids, green for hydrophobic amino acids, and purple for acidic amino acids.

R. erythropolis D‐1 was isolated from a carbendazim‐contaminated farmland in Wenzhou, Zhejiang, China [23]. The cross‐streak method of R. erythropolis D‐1 showed no significant inhibitory effect against 4 model Gram‐positive bacteria, including Streptococcus mutans CGMCC 1.2500, *S*. epidermidis CGMCC 1.160912, Staphylococcus pseudintermedius CICC 10499, and Bacillus subtilis CGMCC 1.821, and 2 model Gram‐negative bacteria including *Salmonella enterica* CGMCC 1.10603 and *Escherichia coli* DH5α (Figure 1a). These findings suggest that R. erythropolis D‐1 does not produce antibiotics or produces trace amounts of antibiotics under laboratory culture conditions, reinforcing the limitation of the classical activity tracking method.

### 2.2 Prediction, Chemical Synthesis and Antibacterial Assay of NRPs From *R. erythropolis* D‐1

Bioinformatic analysis by antiSMASH [24] of the R. erythropolis D‐1 genome (GenBank Accession number: MNP00000000) uncovered numerous BGCs, including 12 NRPS BGCs which accounted for 52.17% of all clusters (Figure 1b and Table S1). Using antiSMASH [25] and PRISM [26], we analyzed four new NRPS BGCs and predicted their corresponding NRP skeletons. However, bioinformatic tools can predict only the amino acid skeletons without predicting cyclization patterns or fatty acid modifications. Starting from the prediction results of the *rezu6* NRPS BGC (GenBank Accession Number: MNPP01000005.1 location 456 760 – 512 881; Figure 1c, Figure S1 and Table S2), we designed and chemically synthesized a preliminary NRP analog library, including linear peptides, cyclic peptides with different cyclization sites, and peptides carrying fatty acids (Figure 1d and Table S3).

Bioactivity assessment of ZURJC1‐8 indicated that only two cyclic peptides ZURJC5 and ZURJC6 displayed antibacterial activity against Gram‐positive bacteria, and exhibited no inhibitory effect on Gram‐negative bacteria (Table 1) or cancer cells (data not shown), suggesting that cyclization between the first amino acid residue and the last amino acid residue might play an essential role to the antibacterial activities. Thus, ZURJC5 (Figure S2) was chosen as a template for further SAR studies to discover more potent analogs with enhanced antibacterial potency.

**TABLE 1:** Antimicrobial activities of NRP analogs ZURJC1‐8.

NRP Analogs (µg/mL)	*S. mutans* CGMCC 1.2500	*S. epidermidis* CGMCC 1.16091	*S. enterica* CGMCC 1.10603	*E. coli* DH5α	*S. pseudintermedius* CICC 10499	*B. subtilis* CGMCC 1.821
ZURJC1	>1024	>1024	>1024	>1024	>1024	>1024
ZURJC2	>1024	>1024	>1024	>1024	>1024	>1024
ZURJC3	>1024	>1024	>1024	>1024	>1024	>1024
ZURJC4	>1024	>1024	>1024	>1024	>1024	>1024
ZURJC5	>1024	512	>1024	>1024	>1024	512
ZURJC6	>1024	1024	>1024	>1024	>1024	>1024
ZURJC7	>1024	>1024	>1024	>1024	>1024	>1024
ZURJC8	>1024	>1024	>1024	>1024	>1024	>1024

### 2.3 The SAR Studies of ZURJC5

**FIGURE 2:**
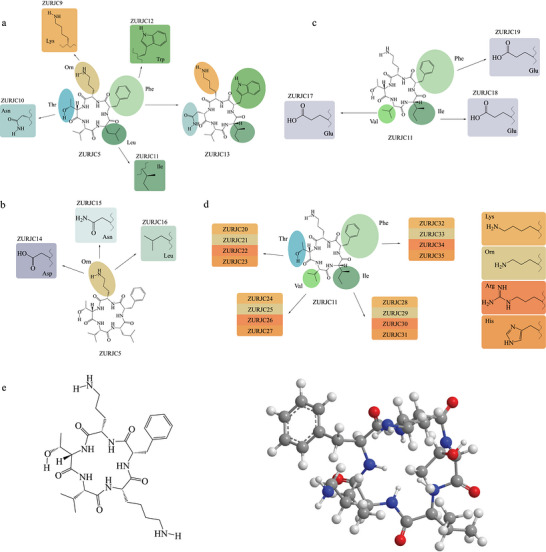
The SAR studies of NRP analogs. (a) The first round of the SAR study. Minor modifications of ZURJC5, including Orn to Lys at position 1 (ZURJC9), Thr to Asn at position 2 (ZURJC10), Leu to Ile at position 4 (ZURJC11), Phe to Trp at position 5 (ZURJC12), and ZURJC13 with all those changes. (b) The second round of SAR study. Amino acid residue alteration at position 1 of ZURJC5, including Orn to Asp (ZURJC14), Asn (ZURJC15), and Leu (ZURJC16). (c) The third round of SAR study. Anionic scan of ZURJC11, including Val to Glu at position 3 (ZURJC17), Ile to Glu at position 4 (ZURJC18), and Phe to Glu at position 5 (ZURJC19). (d) The fourth round of SAR study. Saturated cationic scan of ZURJC11, including Thr to four basic amino acids respectively at position 2 (ZURJC20‐23), Val to four basic amino acids respectively at position 3 (ZURJC24‐27), Ile to four basic amino acids respectively at position 4 (ZURJC28‐31), and Phe to four basic amino acids respectively at position 5 (ZURJC32‐35). (e) Chemical structure and ball‐and‐stick model of ZURJC28.

Since the cyclic peptide ZURJC5 exhibited weak antibacterial activity, we rationally designed a set of analogs based on ZURJC5 (Table S3). The first round of the SAR study was based on minor modifications to the chemical skeleton of ZURJC5 by replacing amino acid residues with other amino acid residues with similar chemical properties, including Orn to Lys at position 1 (ZURJC9), Thr to Asn at position 2 (ZURJC10), Leu to Ile at position 4 (ZURJC11), Phe to Trp at position 5 (ZURJC12), and ZURJC13 with all those changes (Figure 2a). Among these analogs, ZURJC11 displayed a minimum inhibitory concentration (MIC) of 64 µg/mL against *S. epidermidis* CGMCC 1.16091 and *S. pseudintermedius* CICC 10499 (Table 2 and Figure S3). Interestingly, although ZURJC11 showed much better activity comparation of ZURJC5 (eightfold different of MIC), the structures of two molecules are very similar (substitution of Leu to Ile at position 4).

**TABLE 2:** Antibacterial activities of NRP analogs ZURJC9‐37.

NRP Analogs (µg/mL)	*S. mutans* CGMCC 1.2500	*S. epidermidis* CGMCC 1.16091	*S. enterica* CGMCC 1.10603	*S. pseudintermedius* CICC 10499	*B. subtilis* CGMCC 1.821	*E. faecium* CICC 24252	*B. subtilis* CICC 25217
ZURJC9	>128	>128	>128	>128	>128	ND^a^	ND
ZURJC10	>128	128	>128	64	>128	ND	ND
ZURJC11	>128	64	>128	64	128	ND	ND
ZURJC12	>128	>128	>128	>128	>128	ND	ND
ZURJC13	>128	>128	>128	>128	>128	ND	ND
ZURJC14	>128	>128	>128	>128	>128	ND	ND
ZURJC15	>128	>128	>128	>128	>128	ND	ND
ZURJC16	>128	>128	>128	>128	>128	ND	ND
ZURJC17	>128	>128	>128	>128	>128	ND	ND
ZURJC18	>128	>128	>128	>128	>128	ND	ND
ZURJC19	>128	64	>128	128	128	ND	ND
ZURJC20	>128	32	>128	64	64	ND	ND
ZURJC21	>128	64	>128	64	128	ND	ND
ZURJC22	>128	16	>128	16	16	ND	ND
ZURJC23	>128	32	>128	64	64	ND	ND
ZURJC24	>128	16	>128	16	64	ND	ND
ZURJC25	>128	>128	>128	>128	>128	ND	ND
ZURJC26	>128	>128	>128	>128	>128	ND	ND
ZURJC27	>128	8	>128	16	32	ND	ND
ZURJC28	>128	4	>128	8	16	32	8
ZURJC29	>128	>128	>128	>128	>128	ND	ND
ZURJC30	>128	>128	>128	>128	>128	ND	ND
ZURJC31	>128	8	>128	16	32	ND	ND
ZURJC32	>128	32	>128	64	>128	ND	ND
ZURJC33	>128	32	>128	32	128	ND	ND
ZURJC34	>128	8	>128	16	64	ND	ND
ZURJC35	>128	32	>128	32	128	ND	ND
ZURJC36	>128	>128	>128	128	>128	ND	ND
ZURJC37	>128	>128	>128	128	>128	ND	ND

^a^ ND: not determined.

Furthermore, we performed the second round of the SAR study on the amino acid at position 1 of ZURJC5 (Figure 2b), in which the initial basic amino acids were individually replaced with acidic (ZURJC14), neutral hydrophilic (ZURJC15), or hydrophobic (ZURJC16) amino acids. The replacement of the position 1 residue abolished antibacterial activity in all the analogs (Table 2), suggesting that the presence of basic amino acid residues at position 1 is essential to the activities and implying that the antibacterial mechanism of ZURJC5 and its analogs may be related to cationic properties.

### 2.4 The SAR Studies of ZURJC11

As ZURJC11 exhibited the greatest antibacterial activity in previous SAR studies, we proceeded with further SAR studies and constructed analogs ZURJC17‐37 on the basis of ZURJC11 (Figure 2c,d and Table S3). In the third round of the SAR study, an anionic scan of ZURJC11 was carried out by substituting residues with Glu (Figure 2c), followed by antibacterial activity assessments (Table 2). With the exception of ZURJC19, which had a Glu substitution at position 5, all the anion‐scanned analogs lost their antibacterial activity, reinforcing the hypothesis that the peptide's antibacterial mechanism is closely associated with its cationic properties.

Therefore, a saturated cationic scan was conducted on ZURJC11 in the fourth round of the SAR study (Figure 2d). Cationic scanning yielded multiple analogs with stronger antibacterial activity. Among these analogs, ZURJC28 (replacement of Ile with Lys at position 4 of ZURJC11) exhibited the strongest activity (MIC of 4 g/mL against *S. epidermidis* CGMCC 1.16091) (Table 2, Figure 2e and Figure S4). Meanwhile, ZURJC37, which was derived from ZURJC28 with fatty acid addition, completely lost its antibacterial activity, indicating that the mechanism of ZURJC28 is unrelated to the fatty acid moiety. The results of both anionic and cationic scans strongly supported that the antibacterial mechanism of the peptides is predominantly tied to their cationic properties.

### 2.5 Multiple Antibacterial Assays of ZURJC28

Additional MIC tests of ZURJC28 showed MICs of 32 µg/mL against vancomycin‐resistant *Enterococcus faecium* CICC24252 and 8 µg/mL against multidrug‐resistant *Bacillus subtilis* CICC25217 (resistant to erythromycin, tetrazolium red, polymyxin B) (Table 2).

**FIGURE 3:**
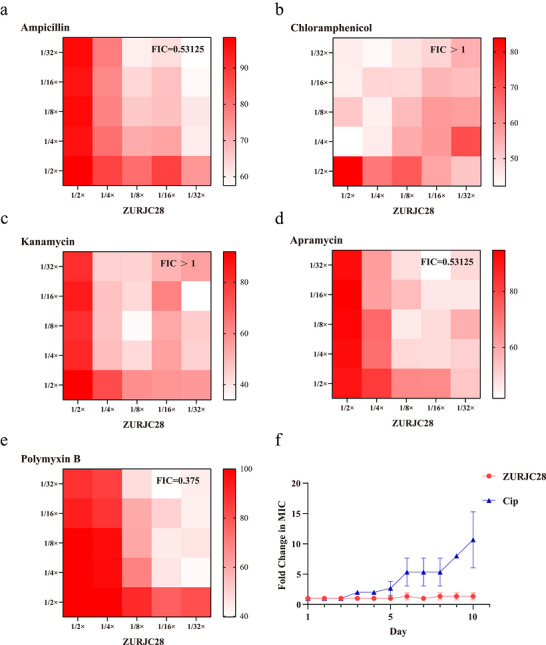
Checkerboard assays and resistance development studies of ZURJC28 against *S. epidermidis* CGMCC 1.16091. (a‐e) Synergistic antibacterial activity of ZURJC28 with ampicillin (a), chloramphenicol (b), kanamycin (c), apramycin (d), and polymyxin B (e). The concentration of ZURJC28 and antibiotics is expressed as different fold of MIC, and the darker red color indicates the higher antibacterial activity. All experiments were performed in technical triplicate (n = 3) and repeated three times independently (n = 3). (f) Resistance development under 0.5‐fold MIC of ZURJC28 and ciprofloxacin during 10 days of serial passages. All experiments were performed in technical triplicate (n = 3) and repeated three times independently (n = 3). Data show the mean of 3 independent replicates ± SD.

Checkerboard assays were conducted with *S. epidermidis* CGMCC 1.16091 as the test strain to evaluate the adjuvant potency of ZURJC28 in association with various antibiotics (Figure 3a‐e). We found that ZURJC28 displayed no synergistic activity when in combination with chloramphenicol or kanamycin, and FICs with apramycin and ampicillin were 0.53125, suggesting additive effects. Interestingly, ZURJC28 potentiated activity of polymyxin B, with an FIC of 0.375. Polymyxin B is a broad‐spectrum antibiotic with strong activity against Gram‐negative bacteria and weak activity against Gram‐positive bacteria. The combination of ZURJC28 and polymyxin B reduced the MIC of polymyxin B against Gram‐positive bacteria by fourfold, indicating the effective synergy of ZURJC28 with polymyxin B.

We assessed the resistance of ZURJC28 against *S. epidermidis* CGMCC 1.16091 and no significant increase in resistance (i.e., MIC fold change ≤ 2) was observed during short‐term (10 days) serial passage (Figure 3f).

### 2.6 Membrane Damage Caused by ZURJC28 Within Several Minutes

**FIGURE 4:**
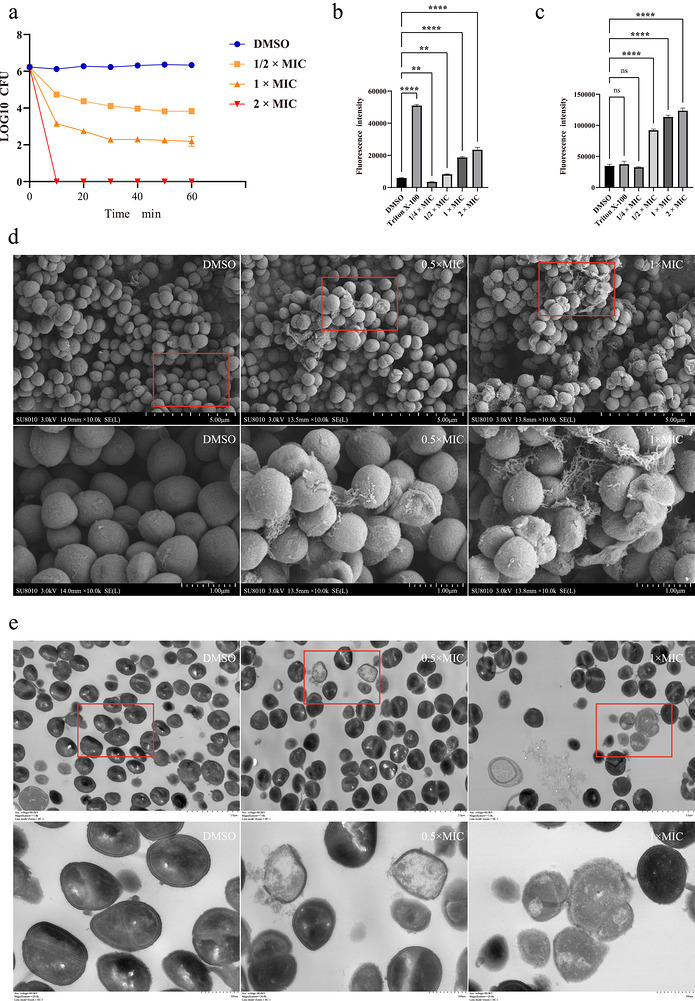
Mode of action for ZURJC28. (a) The time‐dependent killing curve analysis of ZURJC28 against *S. epidermidis* CGMCC 1.16091. *S. epidermidis* CGMCC 1.16091 was exposed to different fold MIC of ZURJC28. Dimethyl sulfoxide (DMSO) was used as negative control. All experiments were performed in technical triplicate (n = 3) and repeated three times independently (n = 3). Data show the mean of 3 independent replicates ± SD. (b, c) Depolarization (b) and disruption (c) of cytoplasmic membrane in *S. epidermidis* CGMCC 1.16091 determined by monitoring the fluorescence intensity of DiSC3‐(5) and PI. Triton X‐100 and DMSO were used as positive and negative controls, respectively. All experiments were performed in technical triplicate (n = 3) and repeated three times independently (n = 3). Data show the mean of 3 independent replicates ± SD. Differences among the groups were analyzed using one‐way ANOVA. **: *p* ≤ 0.01, ****: *p* ≤ 0.0001, ns: *p* > 0.05. (d, e) SEM (d) and TEM (e) images of *S. epidermidis* CGMCC 1.16091 treated with different concentrations of ZURJC28. DMSO was used as control.

*S. epidermidis* CGMCC 1.16091 was used as the indicator strain to elucidate the antibacterial mechanism of ZURJC28. The time‐dependent killing curve analysis of ZURJC28 was first performed to characterize its bactericidal activity (Figure 4a). Remarkably, at 1‐ and 2‐fold MICs, ZURJC28 reduced the number of bacteria by approximately four orders of magnitude within 10–20 min. At a twofold MIC, ZURJC28 displayed rapid bactericidal effects within 10 min. Owing to the rapid and potent bactericidal activity observed, we postulated that ZURJC28 exerts its antibacterial activity by targeting the bacterial cytoplasmic membrane.

The fluorescent probes 3,3’‐dipropylthiadicarbocyanine iodide [DiSC3‐(5)] and propidium iodide (PI) were used to assess the potential and integrity of cytoplasmic membrane. DiSC3‐(5) is a positively charged fluorescent probe that can accumulate in the cytoplasmic membrane, which leads to the fluorescence quenching [27]. When the cytoplasmic membrane is disrupted, DiSC3‐(5) is released into the extracellular solution, increasing the fluorescence intensity. After the bacteria were treated with ZURJC28 for 10 min, the fluorescence intensity of DiSC3‐(5) markedly increased in a dose‐dependent manner (Figure 4b), indicating that ZURJC28 rapidly caused depolarization of the bacterial cytoplasmic membrane.

PI cannot cross the intact cytoplasmic membrane of viable cells and displays weak fluorescence intensity when free in the extracellular milieu [28]. Upon disruption of cytoplasmic membrane, PI can enter the cell through sufficiently large pores and specifically bind to nucleic acids to increase fluorescence intensity. Similarly, after 10 minutes of ZURJC28 treatment, the fluorescence intensity of PI significantly increased in a dose‐dependent manner (Figure 4c), revealing that ZURJC28 caused substantial disruption and pores in the cytoplasmic membrane within 10 min. Compared with ZURJC28, Triton X‐100 acted much slower and failed to show an effective effect under identical conditions.

After *S. epidermidis* CGMCC 1.16091 was incubated with ZURJC28 for 10 min, we immediately fixed the bacterial cells and observed the morphological changes of cytoplasmic membrane by scanning electron microscopy (SEM; Figure 4d). The SEM micrographs clearly revealed deformation, perforation and cytoplasmic leakage of cells exposed to 0.5‐fold MIC ZURJC28, whereas the DMSO control resulted in no apparent damage. When the concentration of ZURJC28 was increased to onefold MIC, the cell damage intensified, and widespread rupture occurred.

The images of transmission electron microscopy (TEM; Figure 4e)revealed distinct membrane fragmentation and discontinuity in the 0.5‐fold MIC ZURJC28‐treated cells, along with leakage of cytoplasmic contents and internal structural collapse. At onefold MIC, the cytoplasmic membrane was completely blurred or absent with massive efflux of cellular content. Collectively, the fluorescence intensity and electron microscopy results illustrate that ZURJC28 directly acts on the cytoplasmic membrane of *S. epidermidis* CGMCC 1.16091, causing large pores and membrane damage within a short time.

### 2.7 Characterization of Interaction Between ZURJC28 and Cytoplasmic Membrane Components

**FIGURE 5:**
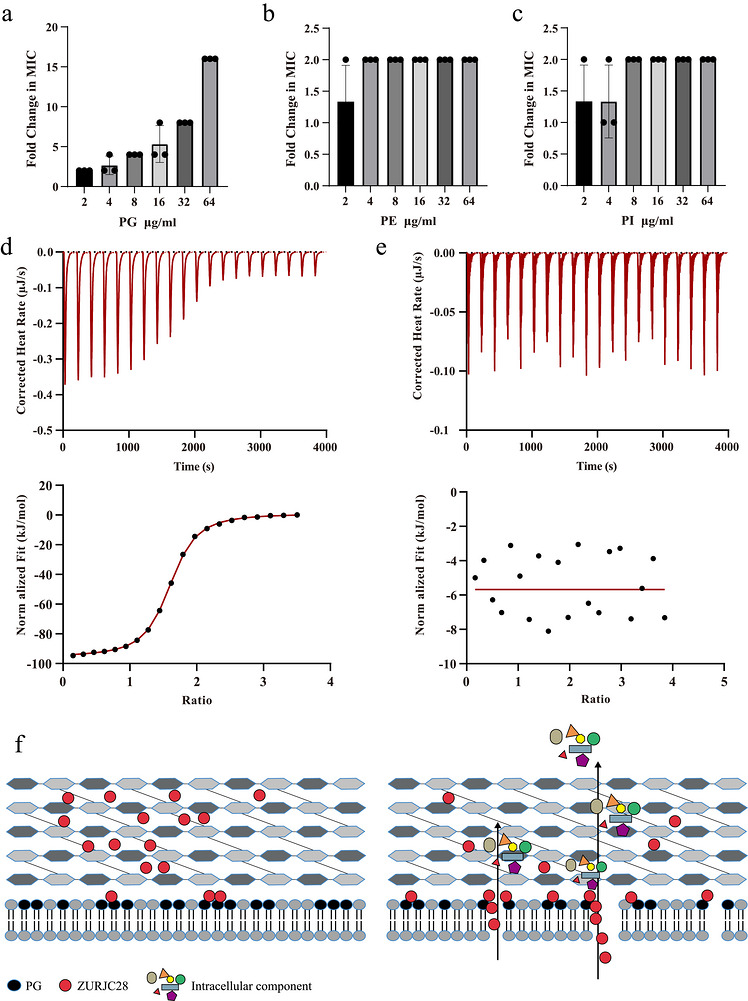
Interaction of ZURJC28 with PG. (a‐c) Fold changes in the MICs of ZURJC28 with exogenous addition of PG (a), PE (b) and PI (c) against *S. epidermidis* CGMCC 1.16091. All experiments were performed in technical triplicate (n = 3) and repeated three times independently (n = 3). (d, e) ITC data for ZURJC28 (d) or its inactive analog ZURJC18 (e) interacting with PG. f) Diagram of the proposed antibacterial mechanism of ZURJC28. ZURJC28 binds the cytoplasmic membrane phospholipid PG in Gram‐positive cell (left). The presence of ZURJC28 causes membrane damage and leakage of intracellular components (right).

The cytoplasmic membrane of Gram‐positive bacteria is rich in phosphatidylglycerol (PG) and contains some phosphatidylethanolamine (PE), whereas phosphatidylinositol (PI) mainly exists in eukaryotic cells [29]. To investigate potential targets of ZURJC28, exogenous PG, PE and PI were added at different concentrations in MIC analysis (Figure 5a‐c). Exogenous addition of PE or PI did not significantly affect the MIC of ZURJC28, and the minor increase in MIC possibly resulted from complex metabolic interactions. In contrast, the exogenous addition of PG led to a dose‐dependent effect on the MIC. When the concentration of PG was increased to 64 µg/mL, the MIC of ZURJC28 increased 16‐fold, suggesting that ZURJC28 likely interacts with PG.

Isothermal titration calorimetry (ITC) was subsequently conducted to confirm the molecular binding between ZURJC28 and PG (Figure 5d and Table S4). Consistent with the results of the lipids assays, we observed that ZURJC28 binds PG at a molar ratio of 1:1.61 (*Kd* = 3.58 × 10^−7 ^M), whereas a representative inactive analog (ZURJC18) from the previous synthesis studies did not bind PG (Figure 5e). On the basis of these findings and those of previous studies, we propose that ZURJC28 exerts its antibacterial activity by binding to PG‐enriched Gram‐positive membranes, disrupting the organization of phospholipid bilayer, which leads to membrane damage and pore formation. The damage then induced changes in membrane and greatly increased permeability, thereby causing rapid leakage of ions, molecules and proteins, ultimately resulting in cell death (Figure 5f).

### 2.8 Metabolomics Analysis Reveals the Deep Antibacterial Mechanism of ZURJC28

Some NRP antibiotics act through multiple mechanisms to prevent bacteria from developing rapid resistance [30]. Our previous results demonstrated that the predominant antibacterial mechanism of ZURJC28 involves disruption of the cytoplasmic membrane by combining with PG. Subsequently, we employed metabolomics to explore whether ZURJC28 has other antibacterial mechanism. The cells of *S. epidermidis* CGMCC 1.16091 were exposed to ZURJC28 and ZURJC18 for 30 min respectively, followed by metabolomics analysis (Figure S5).

**FIGURE 6:**
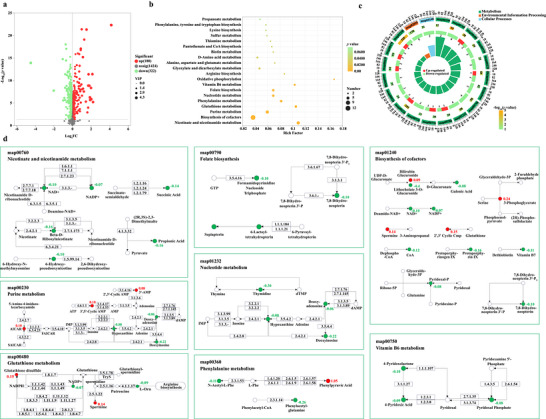
Metabolomics analysis of ZURJC28. (a) The volcano plot of key differential metabolites between ZURJC28‐treated group and ZURJC18‐treated group (VIP>1, *p*<0.05). The horizontal axis represents the fold change in differential expression (Log_2_ FC) and the vertical axis represents the significance of the difference in metabolite expression (‐Log_10_ *p* value). Upregulated genes are shown as red dots, downregulated genes are shown as green dots. (b) The bubble diagram of KEGG enrichment pathways (top 20). The horizontal axis represents the Rich Factor (num‐in‐study / num‐in‐pop), the vertical axis represents the KEGG pathways. The size of bubbles represents the amount of compounds enriched in the metabolic category, the color of bubbles indicates the magnitude of *p* value. (c) The enrichment circle, which has four circles from the outside to the inside. The first circle is the classification of pathways, the area outside the circle is the coordinate scale for the number of compounds, different colors represent different pathway classifications, and the text indicates the specific pathways. The second circle is the number of compounds and *p* value. The third circle is the bar plot of metabolite ratios. Upregulated metabolite ratios are shown as red bars, downregulated metabolite ratios are shown as green bars. The fourth circle is the Rich Factor for each classification. (d) Summary of the significantly enriched pathways. KEGG enrichment pathways with *p* value < 0.005 were selected for detailed analysis.

The volcano plot of differential metabolites showed a pronounced downregulation of lithocholate 3‐O‐glucuronide and upregulation of nelfinavir analogs (Figure 6a). Lithocholate 3‐O‐glucuronide has a disruptive effect on cytoplasmic membrane [31], and its marked downregulation implies that an adaptive response aimed at limiting membrane‐damaging metabolites occurs in bacterial cells to preserve membrane integrity. On the other hand, the upregulation of hydrophobic nelfinavir analog reflects an induced stress response, in which bacterial cells attempt to stabilize the membrane with hydrophobic components.

The KEGG (Kyoto Encyclopedia of Genes and Genomes) enrichment analysis revealed several metabolic pathways that were markedly affected by ZURJC28 (Figure 6b), and those significantly enriched (*p* < 0.005) were further summarized and analyzed (Figure 6c, d), including nicotinate and nicotinamide metabolism, biosynthesis of cofactors, purine metabolism, glutathione metabolism, phenylalanine metabolism, nucleotide metabolism, folate biosynthesis, and vitamin B6 metabolism. Among these significantly enriched pathways, disturbances in nicotinate and nicotinamide metabolism and biosynthesis of cofactors may disrupt electron transport chain [32, 33]; downregulation of deoxyinosine within purine metabolism may reflect impaired DNA/RNA precursor synthesis [34]; and dysregulation of vitamin B6 metabolism and other pathways may imply restricted acyl‐transfer and carbon backbone metabolism [35]. The metabolomics results may represent secondary downstream consequences of rapid membrane damage, which could be presented as evidence of global metabolic perturbation following membrane disruption.

### 2.9 ZURJC28 Shows a Preliminary in Vitro Safety Profile

**FIGURE 7:**
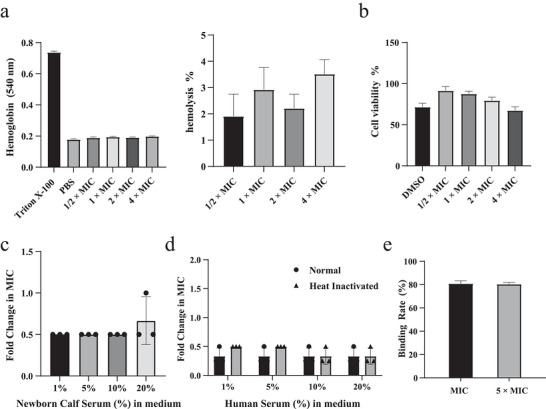
In vitro safety assays of ZURJC28. (a) Hemolytic activity of ZURJC28 against rabbit red blood cells. Triton X‐100 and DMSO were used as positive and negative controls, respectively. All experiments were performed in technical triplicate (n = 3) and repeated three times independently (n = 3). Data show the mean of 3 independent replicates ± SD. (b) Cytotoxic effects of ZURJC28 against THLE‐2 cells. DMSO was used as negative control. All experiments were performed in technical triplicate (n = 3) and repeated three times independently (n = 3). Data show the mean of 3 independent replicates ± SD. (c) Newborn calf serum binding assay of ZURJC28. All experiments were performed in technical triplicate (n = 3) and repeated three times independently (n = 3). Data show the mean of 3 independent replicates ± SD. (d) Human serum and heat inactivated human serum binding assays of ZURJC28. All experiments were performed in technical triplicate (n = 3) and repeated three times independently (n = 3). Data show the mean of 3 independent replicates ± SD. (e) The plasma protein binding assay of ZURJC28 at two different concentrations. All experiments were performed in technical triplicate (n = 3) and repeated three times independently (n = 3). Data show the mean of 3 independent replicates ± SD.

Since PG is rare in mammalian cell membranes, ZURJC28 may have low toxicity in humans, and we further examined hemolytic and cytotoxic properties of ZURJC28 (Figure 7a, b). Compounds with a hemolysis rate less than 5% are generally considered non‐hemolytic, and ZURJC28 showed hemolysis rates less than 5% at all tested concentrations. Meanwhile, cell viability of ZURJC28 remained 67.2% at fourfold MIC and increased to 87.4% at onefold MIC, demonstrating that ZURJC28 has no significant hemolytic or cytotoxic effects.

We also evaluated the serum stability and plasma protein binding of ZURJC28. We first conducted a serum binding assay of newborn calf serum. The results showed that the MIC of ZURJC28 was reduced to 0.5‐fold (Figure 7c). Human serum contains components of the complement system, which can be inactivated by exposure to elevated temperatures. To investigate whether the decrease in the MIC is related to the complement system, we separately performed serum binding assays of human serum and heat inactivated human serum. Both exogenous additions reduced the MIC of ZURJC28 to 0.25‐fold (Figure 7d), indicating that the increase in antibacterial activity is independent of complement system. In the plasma protein binding assay, we observed that the protein binding rate of ZURJC28 was over 80% (Figure 7e). Together with the membrane‐disruptive activity of ZURJC28, we hypothesize that ZURJC28 may exert synergistic effects with immunoglobulins.

## 3 Conclusions

The discovery and development of natural product antibiotics have substantially reduced the mortality caused by bacterial infections. However, the rising antibiotic resistance of bacteria has become a major challenge in clinical medicine [36]. In this study, we initially attempted to isolate antibiotics from *R. erythropolis* D‐1 by activity tracking method. However, we didn't find any antibiotics, which may be due to the lack of antibiotic production under laboratory culture conditions or silent BGCs. The syn‐BNP discovery approach accelerates antibiotic development timelines, but it can yield only antibiotic analogs that may have lower activity than natural antibiotics. We applied syn‐BNP discovery approach to analyze the genome of R. erythropolis D‐1 and chemically synthesized eight initial NRP analogs. Nonetheless, the antibacterial activities of these analogs were weak, likely because they are analogs of natural antibiotics or because the natural antibiotics themselves have low activity.

SAR studies have previously been applied to the development of analogs of natural antibiotics or to elucidate compound mechanisms [37, 38]. Through four rounds of SAR studies, we found that basic amino acids are especially important in analogs. Position 1 for cyclization must be a basic amino acid, and the cationic scan substantially improved the antibacterial potency of analogs, whereas the anionic scan reduced it. These results imply that the antibacterial mechanism of our NRPs is associated with their cationic properties. Our SAR optimization enhanced the antibacterial activity of the analogs against *S. epidermidis* CGMCC 1.16091 from MIC 512 to 4 µg/mL, resulting in a 128‐fold improvement (Tables 1 and 2). The enhanced activity conferred by the addition of basic amino acids supports that the molecules act on negatively charged membrane components, which aligns with the results of mechanistic studies. We observed that combining the syn‐BNP discovery approach with SAR studies integrates the time‐saving advantages of syn‐BNP discovery approach and the activity‐enhancing strengths of SAR studies, facilitating rapid discovery of novel and highly active antibiotic analogs.

Antibiotic mechanisms vary widely. For example, ciprofloxacin targets DNA gyrase and topoisomerases [39]; erythromycin binds the 50S ribosomal subunit to inhibit protein synthesis [40]; nitrofurantoin forms intermediates that attack diverse cellular components nonspecifically [41]; and daptomycin disrupts membranes by calcium‐dependent interactions [42]. The mechanism of cationic NRPs typically involves membranes. Our assays showed that ZURJC28 binds PG, inducing membrane damage and rapid cell lysis. This mechanism is similar to that of daptomycin, but ion‐dependence experiments (Figure S6) revealed that the activity of ZURJC28 is independent of calcium ion. Protein‐targeting antibiotics are easily circumvented through single point mutations of proteins [43], whereas the membrane‐targeting “physical” action makes it difficult for bacteria to develop resistance, which may account for the low resistance of daptomycin and ZURJC28.

Overall, our study presents an NRP analogs discovery strategy that combines syn‐BNP discovery approach with SAR studies, which improves the structure prediction limitations of syn‐BNP discovery approach. We report ZURJC28, an NRP analog that targets PG‐enriched Gram‐positive membranes and rapidly inhibits Gram‐positive bacteria. Compared with natural products, predicted NRPs generally exhibit lower activity owing to structural divergence. However, our strategy can enhance analog activity and compensate for this limitation. However, both syn‐BNP discovery approach and SAR studies depend on bioinformatics accuracy, which requires further algorithm development. Recent advances in artificial intelligence may improve bioinformatics accuracy. Moreover, our current work is limited to in vitro assays and lacks animal infection models. The mechanism by which serum reduces the MIC of ZURJC28 still requires further experimentation. Future research will include more detailed mechanistic exploration and animal infection models of ZURJC28 to elucidate its clinical applicability.

## 4 Experimental Section/Methods

### 4.1 Strains and Media

*S. mutans* CGMCC 1.2500, *S. epidermidis* CGMCC 1.16091, *S. enterica* CGMCC 1.10603, and *B. subtilis* CGMCC 1.821 were purchased from the China General Microbiological Culture Collection Center (CGMCC). *S. pseudintermedius* CICC 10499, *E. faecium* CICC24252, and *B. subtilis* CICC25217 were purchased from the China Center of Industrial Culture Collection (CICC). *R. erythropolis* D‐1 was isolated by our laboratory. For antibacterial activity assays, *B. subtilis* CICC25217 was cultured in Luria Bertani (LB) medium at 37°C and 220 rpm, and other strains were cultured in Nutrient Broth (NB) medium at 30°C and 220 rpm. For time‐dependent killing assay, *S. epidermidis* CGMCC 1.16091 was cultured in Trypticase Soy Broth (TSB) medium at 30°C and 220 rpm for overnight growth of the strain. And the bacterial suspension was diluted in Cation Adjusted Mueller Hinton Broth (CAMHB) medium and incubated at 30°C to reach the required bacterial density for the assay rapidly. For ion‐dependence experiments, *S. epidermidis* CGMCC 1.16091 was cultured in Mueller Hinton Broth (MH) medium at 30°C and 220 rpm for creating a low calcium ion environment. Unless otherwise specified, all chemicals and media were purchased from Solarbio.

### 4.2 Bioinformatic Analysis and Chemical Synthesis of Peptides

AntiSMASH was used to analyze the genome of *R. erythropolis* D‐1. For the *rezu6* NRPS BGC, the 10 active site residues (Stachelhaus code) were identified using antiSMASH and the peptide skeletons were predicted using antiSMASH and PRISM [24, 25, 26]. The chemical structure of the putative peptides was mapped by ChemDraw software. Peptides were synthesized by GL Biochem (Shanghai, China) using solid phase peptide synthesis (SPPS). The Liquid Chromatography‐Mass Spectrometry (LC‐MS) analyses and accurate molecular weights of peptides were also determined by GL Biochem. The purity of all the synthesized peptides was above 95%.

### 4.3 The MIC Assay

The MIC assay of all the synthesized peptides was conducted through micro‐broth dilution method according to the Clinical and Laboratory Standards Institute (CLSI) [44, 45]. All peptides were dissolved in DMSO to give a concentration of 12.8 mg/mL and were determined at concentrations ranging from 128 to 1 µg/mL. Apramycin and DMSO were used as positive and negative controls, respectively. The bacterial strain was inoculated at a 0.1% concentration into the fresh medium and cultured overnight. Subsequently, the bacterial suspension was diluted to a density of 5 × 10^5^ colony‐forming units (CFUs) mL^−1^ and 90 µL of this solution was added to each 96‐well plate and mixed with 10 µL of peptide solution at different concentrations. Then, the 96‐well plates were incubated at 30°C or 37°C and 220 rpm for 16 h. The MIC value was recorded as the minimum peptide concentration without visible bacterial strain, based on the optical density (OD) values of 600 nm using TECAN Spark microplate reader.

### 4.4 Cytotoxicity Assay

The cytotoxicity of all the synthesized peptides was tested using the Cell Counting Kit‐8 (CCK8; Solarbio) [46]. HT29 and THLE‐2 cells were seeded in 96‐well plates at a density of 1 × 10^4^ cells/well in medium containing 90% Dulbecco's Modified Eagle Medium (DMEM), 10% fetal bovine serum and 1% Penicillin/Streptomycin at 37°C for 24 h with 5% CO_2_. Then the peptides at concentrations ranging from 128 to 1 µg/mL were added to each well. After 24 h of incubation, the medium was removed and fresh medium supplemented with 10% CCK‐8 reagent was added, followed by 2 h incubation. The DMSO was used as negative control and the OD values at 450 nm were measured using TECAN Spark microplate reader. The cell survival rate (%) was calculated according to the formula: Cell survival rate (%) = 100% × (Peptide OD_450_ – Zero OD_450_) / (DMSO OD_450_ – Zero OD_450_).

### 4.5 Membrane Depolarization Assay

The DiSC3‐(5) dye was used to analyze the effect of synthesized peptides treatment on the membrane depolarization of Gram‐positive bacteria [27]. The bacterial strain was cultured overnight, and the cells were subsequently washed three times and resuspended in PBS. The bacterial suspension was then adjusted to an OD_600_ of 0.5 and incubated with 1 µM DiSC3‐(5) and peptides at concentrations ranging from twofold MIC to 0.25‐fold MIC in the dark for 10 min. Triton X‐100 and DMSO were used as positive and negative controls, respectively. Membrane depolarization was monitored by the fluorescence intensity of DiSC3‐(5) (Excitation wavelength = 622 nm, Emission wavelength = 670 nm) using SpectraMax iD5 (Molecular Devices) microplate reader.

### 4.6 Membrane Permeability Assay

The PI dye was used to analyze the effect of synthesized peptides treatment on the integrity of cytoplasmic membrane of Gram‐positive bacteria [28]. The bacterial strain was cultured overnight, and the cells were subsequently washed three times and resuspended in PBS. The bacterial suspension was then adjusted to an OD_600_ of 0.5 and incubated with 1.5 µM PI and peptides at concentrations ranging from twofold MIC to 0.25‐fold MIC in the dark for 10 min. Triton X‐100 and DMSO were used as positive and negative controls, respectively. Membrane integrity was monitored by the fluorescence intensity of PI (Excitation wavelength = 535 nm, Emission wavelength = 615 nm) using SpectraMax iD5 (Molecular Devices) microplate reader.

### 4.7 Time‐Dependent Killing Assay

The bacterial strain was cultured overnight and then adjusted to an OD_600_ of 0.5 with fresh TSB medium. The bacterial suspension was diluted 1:100 in 5 mL fresh CAMHB medium and incubated with peptides at concentrations ranging from twofold MIC to 0.5‐fold MIC. After 10, 20, 30, 40, 50 and 60 min, 100 µL aliquots of cells were removed and diluted 1:10‐1:1 × 10^5^ in fresh TSB medium. The serial dilutions were plated on TSB agar plates and cultured at 37°C overnight. Then the colonies were counted, and the CFUs/mL were calculated. The DMSO was used as negative control.

### 4.8 Checkerboard Assays

The MIC of each antibiotic was tested prior to determining their synergistic antibacterial activity with peptides by micro‐broth dilution method [47]. The bacterial suspension was prepared according to the MIC assay and then incubated with peptide and antibiotics at concentrations ranging from 1/2‐fold MIC to 1/32‐fold MIC in 96‐well plates. After 16 h of incubation, the OD_600_ was measured using TECAN Spark microplate reader. The DMSO was used as negative control. The inhibition rate of each mixture was calculated according to the formula: inhibition rate (%) = 100% × (DMSO OD_600_ – Mixture OD_600_) / (DMSO OD_600_ – Zero OD_600_). The Fractional Inhibitory Concentration (FIC) index was calculated according to the formula: FIC = (MIC of peptide in combination) / (MIC of peptide alone) + (MIC of antibiotics in combination) / (MIC of antibiotics alone). The synergistic effect of peptide and antibiotics was defined as FIC ≤ 0.5, the additive effect was defined as 0.5< FIC ≤ 1, the indifference effect was defined as 1< FIC ≤ 2, and the antagonism effect was defined as FIC > 2.

### 4.9 Bacterial Resistance Development Assays

The bacterial resistance toward synthesized peptide was determined through serial passage in liquid broth [48]. The bacterial strain was inoculated at a 0.1% concentration into the fresh medium and cultured overnight. Subsequently, the bacterial suspension was diluted to a density of 5 × 10^5^ CFU mL^−1^ and 90 µL of this solution was added to each 96‐well plate and mixed with the 10 µL of peptide solution at different concentrations. After 16 h of incubation, the OD_600_ was measured using TECAN Spark microplate reader. The second round of assay was based on the bacterial suspension from 0.5‐fold MIC well. The MIC was determined according to the method described above. This process was repeated for 10 passages.

### 4.10 SEM and TEM

The effects of synthesized peptides on the cytoplasmic membrane of Gram‐positive bacteria were measured by SEM and TEM. The bacterial strain was cultured to the exponential phase and incubated with peptides for 10 min. The cells were subsequently washed three times and resuspended in 2.5% glutaraldehyde. After 24 h of fixation at 4°C, the cell samples were observed using the ZEISS GeminiSEM 300 scanning electron microscope and HT7800 (Hitachi) transmission electron microscope.

### 4.11 Lipids Inhibition Assay

The effects of phospholipids (PG, PE, PI) on the antibacterial activity of synthesized peptide were evaluated using the checkerboard assay. The bacterial suspension was prepared according to the MIC assay and then incubated with peptide and phospholipids at concentrations ranging from 64 to 2 µg/mL in 96‐well plates. After 16 h of incubation, the OD_600_ was measured using TECAN Spark microplate reader. The DMSO was used as negative control.

### 4.12 Isothermal Titration Calorimetry (ITC)

The interaction between synthesized peptides and PG was measured using ITC. Both the peptides and PG were diluted in the same standard buffer system containing 10 mM HEPES, pH 7.4, supplemented with 5% DMSO. The final concentrations were 5 µM PG in the syringe and 1 µM peptides in the sample cell. Before the experiment, each sample was degassed in a vacuum chamber for 10 min to remove the gases. Using the sampling needle of the NANO ITC (TA), 300 µL peptide (1 µM) was loaded into the sample cell as the titrand, and 60 µL PG (5 µM) was drawn into the injection syringe as the titrant. Experimental parameters were set to 25°C with a stirring speed of 250 rpm, high‐gain feedback mode, and a reference power of 5 µcal/s. After an initial equilibration period of 300 s to ensure baseline stability, titrations were conducted using an initial injection of 0.5 µL followed by 20 injections of 2.0 µL at 200 s intervals. To account for background heat from PG dilution, possible hydrolysis, and buffer‐associated effects, PG was titrated into blank buffer under the same experimental conditions. The equilibrium dissociation constant (*Kd*), stoichiometry (*N*), enthalpy (*ΔH*), and entropy (*ΔS*) were analyzed using nanoAnalyzer software (version 3.7). Baseline correction was first applied to remove baseline drift, followed by peak integration to obtain the heat per injection. The binding isotherms were then generated according to the molar ratio and fitted using a one set of sites model to obtain the thermodynamic parameters.

### 4.13 Hemolytic Assay

The hemolytic activity of synthesized peptide was evaluated using rabbit red blood cells [49]. Briefly, 100 µL red blood cell suspension and 900 µL peptide at concentrations ranging from 1/2‐fold MIC to 1/32‐fold MIC were mixed and incubated at 37°C for 1 h. Cells were centrifuged at 1500 rpm for 5 min and the supernatant was removed. The OD_540_ was measured using VarioskanFlash (Thermo Fisher) microplate reader. Triton X‐100 and DMSO were used as positive and negative controls, respectively. The hemolysis rate was calculated according to the formula: Peptide hemolysis rate (%) = 100% × (Peptide OD_540_ – DMSO OD_540_) / (Triton X‐100 OD_540_ – DMSO OD_540_).

### 4.14 Metabolomics Analysis

The bacterial strain was cultured to the exponential phase and incubated with peptides for 30 min. At least six biological replicates of each treatment were extracted and profiled using a UHPLC‐Q Exactive HF‐X system by Majorbio Bio‐Pharm Technology Co., Ltd. (Shanghai, China) [50]. The quality control samples (QCs) were prepared by mixing all the sample metabolites in equal volumes and injected at regular intervals in order to monitor the stability of the analysis. The raw data were processed by Progenesis QI (Waters Corporation, Milford, USA) software. Internal standard peaks and any known false positive peaks (including noise, column bleed, and derivatized reagent peaks) were removed from the data matrix, deredundant and peak pooled. The metabolites were identified by matching the MS and MS/MS mass spectrometry information with the public metabolic database. The major databases were the Human Metabolome Database (HMDB) (http://www.hmdb.ca/), the Metlin (https://metlin.scripps.edu/) and the self‐compiled Majorbio Database (MJDB) of Majorbio. The confidence level of the identification is level 2 according to the Metabolomics Standards Initiative (MSI). The data were then uploaded to the Majorbio free online platform (https://cloud.majorbio.com) to be analyzed.

Among the metabolic characteristics detected in any set of samples, at least 80% were retained. The missing values were filled with the minimum value, and each metabolic signature was normalized to the sum. To reduce the errors, the sum normalization method was used to normalize the response intensities of the sample MS peaks. Variables with relative standard deviation (RSD) > 30% in QC samples were removed and log10 logarithmicized in order to obtain the final data matrix.

The principal component analysis (PCA) and orthogonal least partial squares discriminant analysis (OPLS‐DA) were performed by the R package “ropls” (version 1.6.2) with 7‐cycle interactive validation used to evaluate the robustness of the model. The variable importance in the projection (VIP, obtained by the OPLS‐DA) and the *p* value (generated by Student's t test) were employed to screen the significantly different metabolites (VIP>1, *p*<0.05). The analysis of enrichment pathways was based on KEGG database (http://www. genome.jp/kegg/).

### 4.15 Serum Binding Assay

The serum stability of ZURJC28 was evaluated using the MIC assay. Different concentrations (1%, 5%, 10% and 20%) of newborn calf serum, human serum (purchased from YaJi Biological), or heat inactivated (56°C,30 min) human serum were added to the fresh medium and then incubated with peptide in 96‐well plates at 30°C. After 16 h of incubation, the OD_600_ was measured using TECAN Spark microplate reader. The DMSO was used as negative control.

### 4.16 Human Plasma Protein Binding Assay

Ultracentrifugation method was selected to perform human plasma protein binding assay [51]. Human plasma was collected from healthy volunteers, which was approved by the ethics committee of Changsha Nanya Hospital (IRB approval number: [mix‐CSNY‐PE‐20260310]). Peptide was dissolved in DMSO and spiked into pooled human plasma to achieve final concentrations of 4 and 20 µg/mL. Samples were then incubated for a duration equivalent to the centrifugation time at 37°C to ensure equilibrium. The tubes were centrifuged at 100 000 rpm (∼ 436 000 × g) at 37°C for 2–4 h using Optima ultracentrifuge (Beckman Coulter). The supernatant which contained the unbound fraction was carefully collected subsequently (Cfree). Aliquots of the same plasma samples were incubated under the same conditions without centrifugation, representing the total drug concentration (C0). Samples were analyzed using liquid chromatography‐tandem mass spectrometry (LC‐MS/MS) by AB SCIEX API 7500 mass spectrometer equipped with an electrospray ionization (ESI), source operated in positive ion mode. The protein binding rate was calculated according to the formula: Protein binding rate (%) = 100 – (C0 – Cfree) / C0 × 100%.

### 4.17 Ion‐Dependence Experiments

The ion‐dependence experiments of ZURJC28 and daptomycin were evaluated using the MIC assay. The experiment was conducted under two different concentrations of calcium ions. The initial MH medium provided a low calcium ion environment (0.07 – 0.15 mM) and 1.25 mM CaCl_2_ was added to the fresh medium to create a high calcium ion environment. Peptide and daptomycin were incubated in 96‐well plates at 30°C. After 16 h of incubation, the OD600 was measured using TECAN Spark microplate reader. The DMSO was used as negative control.

### 4.18 Statistical Analysis

Unless otherwise specified, all experiments were performed in technical triplicate (n = 3) and repeated three times independently (n = 3). The values reported are shown as mean ± standard deviation (SD). Statistical analysis was performed using one‐way ANOVA. *p* values are indicated in the figures (**: *p* ≤ 0.01, ****: *p* ≤ 0.0001, ns: *p* > 0.05). Statistical significance in experimental data was determined using GraphPad Prism 9.3.1 software.

## Conflicts of Interest

The authors declare no conflicts of interest.

## Supporting information




**Supporting File**: advs76103‐sup‐0001‐SuppMat.docx.

## Data Availability

The data that support the findings of this study are available from the corresponding author upon reasonable request.
