# Supplementary material for: Identification of a Nonribosomal Peptide Analog With Activity Against Multiple Gram‐Positive Bacteria via a Synthetic Bioinformatic Natural Product Discovery Approach
Source: Adv Sci (Weinh). 2026 Jun 11:e76103. Online ahead of print. doi: 10.1002/advs.76103 (PMC13336364; doi:10.1002/advs.76103)
Supplement: Supplementary file 1 — Supporting File: advs76103‐sup‐0001‐SuppMat.docx. [file ADVS-9999-e76103-s001.docx]

Supporting Information

Identification of a Nonribosomal Peptide Analog with Activity against Multiple Gram-positive Bacteria via a Synthetic Bioinformatic Natural Product Discovery Approach

Keyi Chen, Jiayi Liang, Yujia Wu, Jianan Xu, Yi Liu, Wenguang Wang, Xinhang Jiang, Benjie Gao^*^, Yueyue Wang^*^, Hui Jiang^*^

This Supporting Information includes:

Figure S1 to S6

Table S1 to S4

Figures


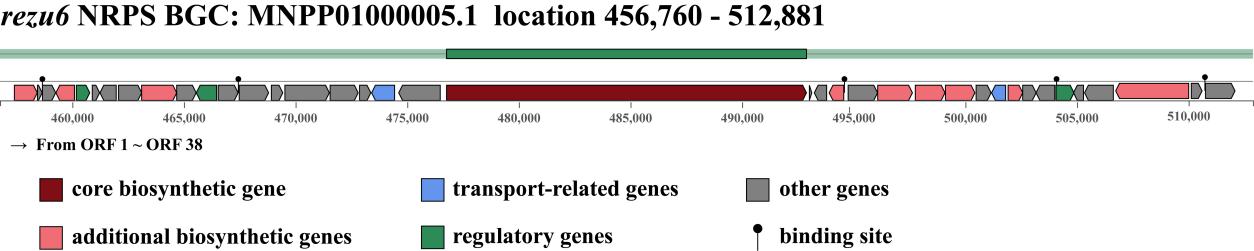


**Figure** S1. The entire NRPS BGC of *rezu6*.


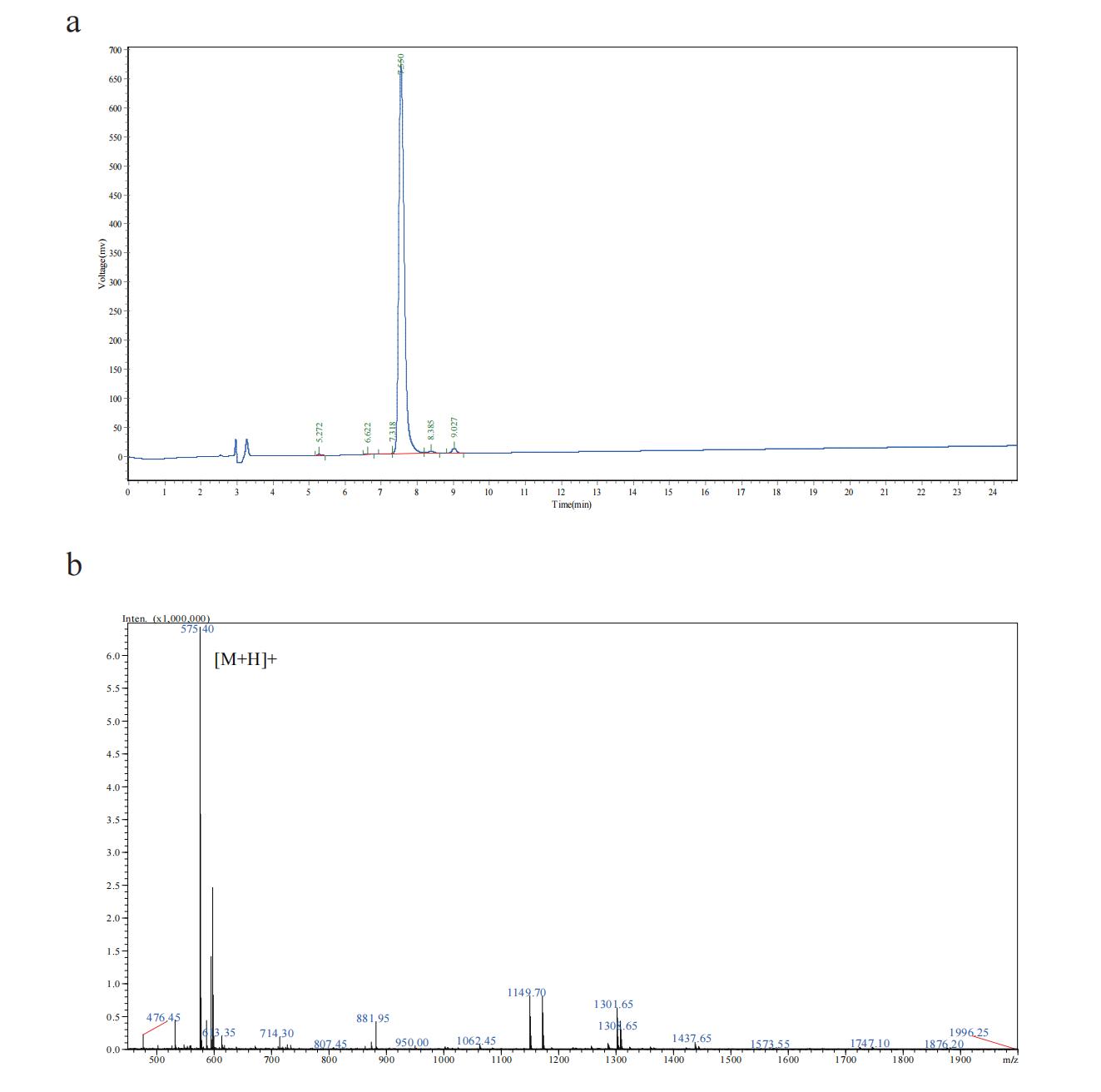


**Figure** S2. LC-MS analysis of ZURJC5. a. HPLC chromatogram of ZURJC5. b. Mass spectra of ZURJC5.


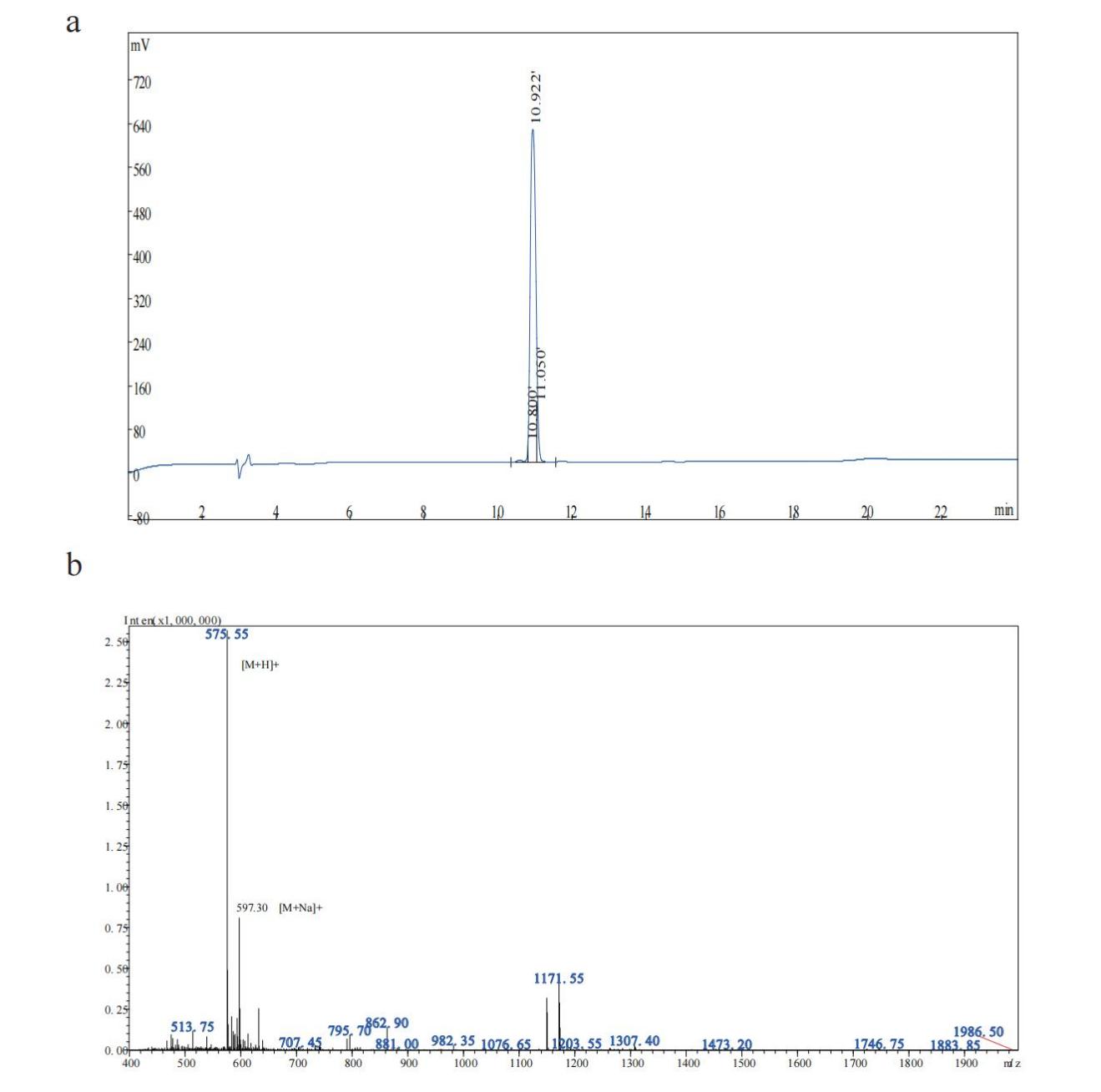


**Figure** S3. LC-MS analysis of ZURJC11. a. HPLC chromatogram of ZURJC11. b. Mass spectra of ZURJC11.


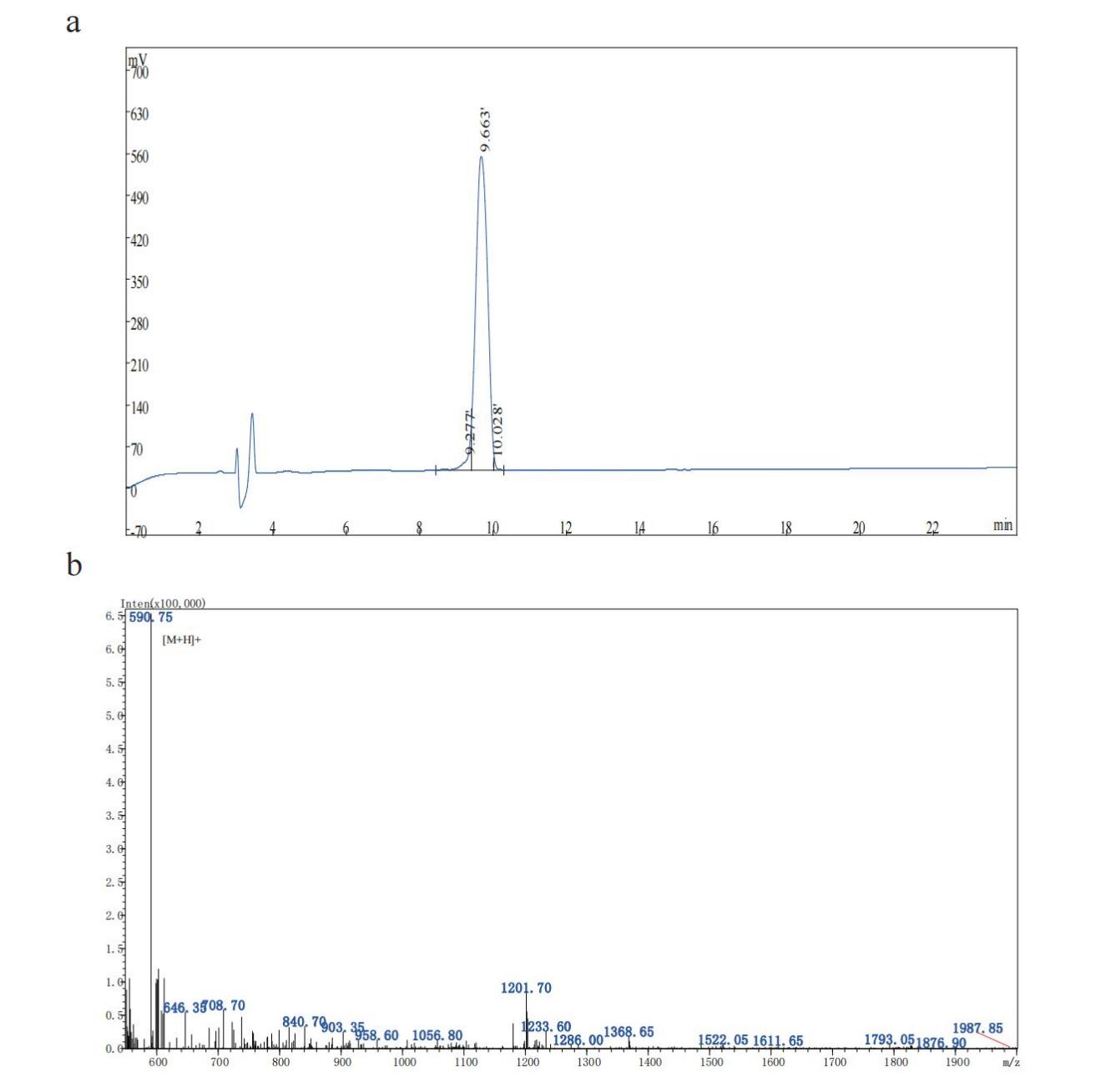


**Figure** S4. LC-MS analysis of ZURJC28. a. HPLC chromatogram of ZURJC28. b. Mass spectra of ZURJC28.


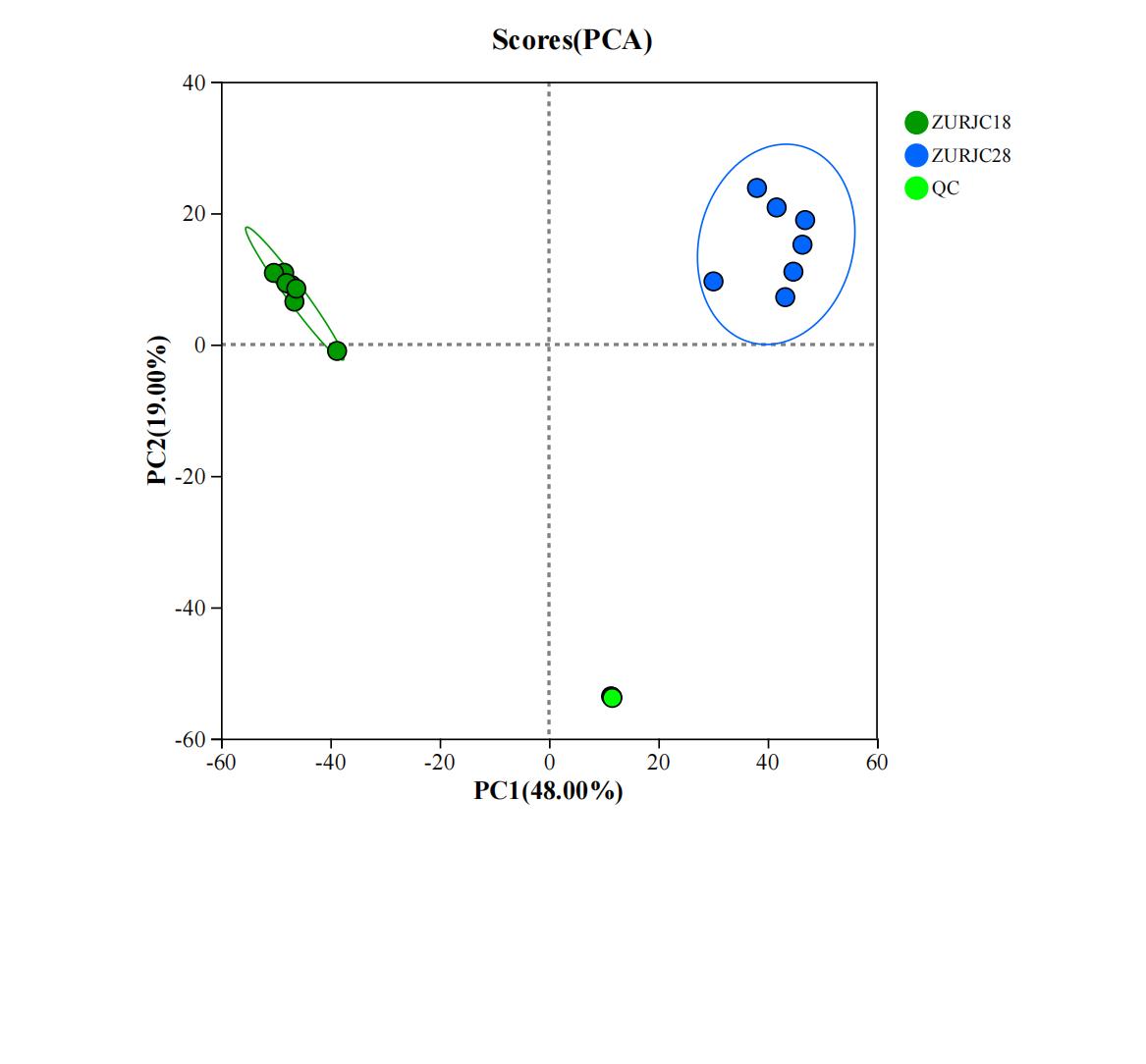


**Figure S5.** Principal component analysis (PCA) plot of ZURJC28 and ZURJC18 in metabolomics analysis.


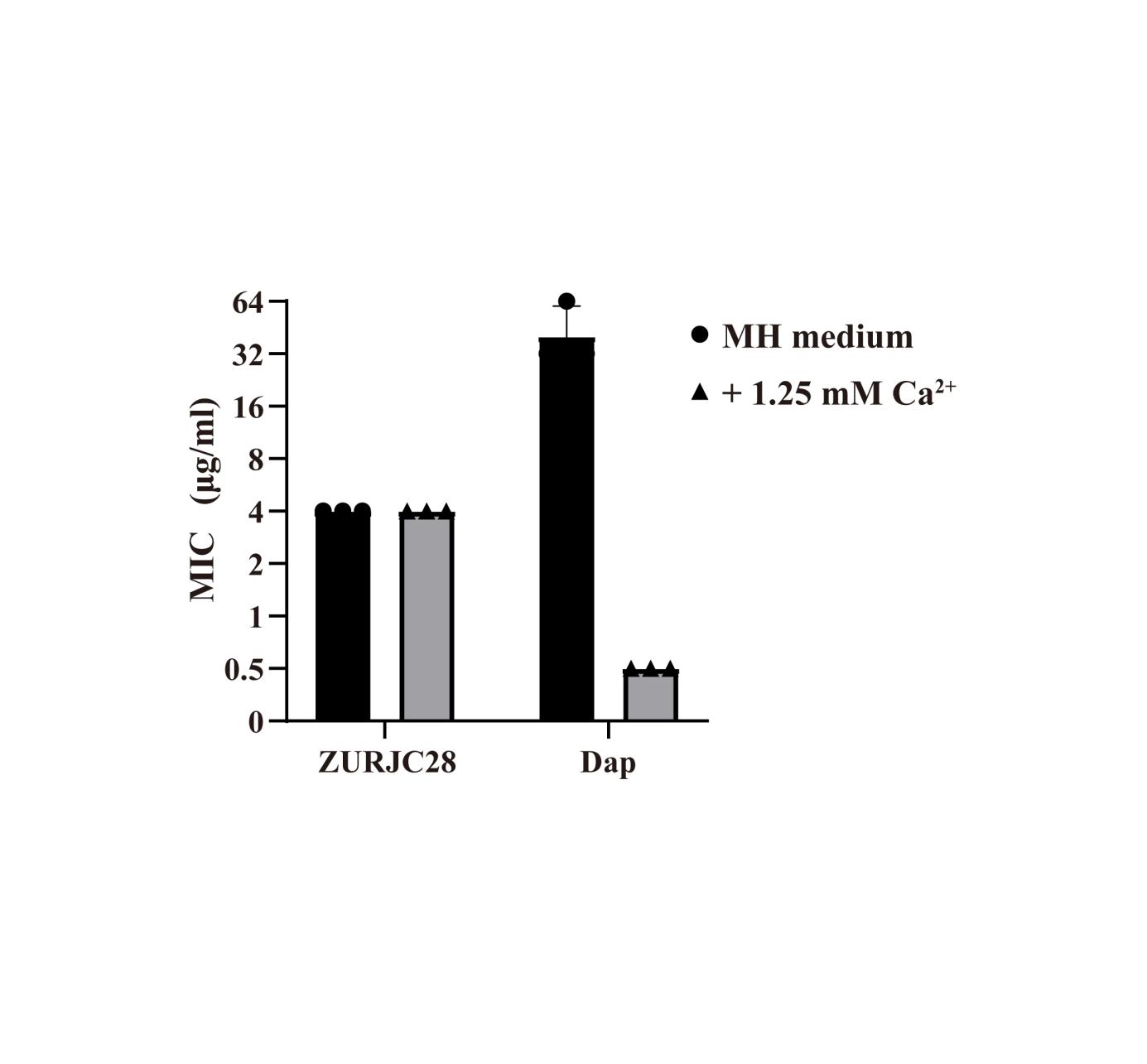


**Figure S6.** Calcium-independent assay of ZURJC28 and daptomycin. All experiments were performed in technical triplicate (n = 3) and repeated three times independently (n = 3). Data show the mean of 3 independent replicates ± SD.

Tables

Table S1. The BGC of *Rhodococcus erythropolis* D-1 genome analyzed by antiSMASH.

| Region | GenBank Accession | Type | Location | Similarity Confidence |
| --- | --- | --- | --- | --- |
| Region 1.1 | MNPP01000011.1 | NRPS | 1 ~ 29,063 | None |
| Region 2.1 | MNPP01000012.1 | NRPS | 1 ~ 38,902 | High |
| Region 2.2 | MNPP01000012.1 | RiPP | 56,086 ~ 78,883 | None |
| Region 3.1 | MNPP01000013.1 | NRPS | 128,581 ~ 168,545 | None |
| Region 5.1 | MNPP01000015.1 | NRPS | 94,777 ~127,414 | None |
| Region 6.1 | MNPP01000016.1 | NRPS | 1 ~ 22,916 | Low |
| Region 23.1 | MNPP01000002.1 | Other | 42,604 ~ 109,180 | Low |
| Region 23.2 | MNPP01000002.1 | Other | 140,316 ~ 194,601 | None |
| Region 23.3 | MNPP01000002.1 | NRPS | 481,129 ~ 539,064 | High |
| Region 23.4 | MNPP01000002.1 | NRPS | 800,677 ~ 843,211 | None |
| Region 28.1 | MNPP01000036.1 | NRPS | 1 ~ 3,058 | None |
| Region 34.1 | MNPP01000003.1 | NRPS | 1 ~ 31,744 | High |
| Region 34.2 | MNPP01000003.1 | NRPS | 369,970 ~ 428,161 | Medium |
| Region 44.1 | MNPP01000004.1 | Other | 188,407 ~ 210,989 | None |
| Region 45.1 | MNPP01000005.1 | Ectoine | 147,580 ~ 157,978 | Medium |
| Region 45.2 | MNPP01000005.1 | Terpene | 356,898 ~ 382,263 | Low |
| Region 45.3^a)^ | MNPP01000005.1 | NRPS | 456,760 ~ 512,881 | None |
| Region 45.4 | MNPP01000005.1 | NRPS | 515,002 ~ 540,370 | None |
| Region 46.1 | MNPP01000006.1 | Butyrolactone | 350,977 ~ 361,801 | None |
| Region 47.1 | MNPP01000008.1 | PKS | 12,937 ~ 57,880 | None |
| Region 47.2 | MNPP01000008.1 | PKS | 193,754 ~ 239,864 | None |
| Region 49.1 | MNPP01000010.1 | RiPP | 11,686 ~ 23,616 | Medium |
| Region 49.2 | MNPP01000010.1 | RiPP | 183,960 ~ 214,056 | None |

^a)^: *rezu6* NRPS BGC.

Table S2. The gene annotations of *rezu6* NRPS BGC (GenBank Accession Number: MNPP01000005.1 location 456,760 - 512,881).

| ORF | Length | | Function | Most Similar Description | Identities |
| --- | --- | --- | --- | --- | --- |
|  | NT | AA |  |  |  |
| 1 | 1029 | 342 | biosynthetic-additional | biotin synthase BioB | 99% |
| 2 | 213 | 70 | other | biotin synthase auxiliary protein BsaP | 89% |
| 3 | 591 | 196 | other | DUF2567 domain-containing protein | 94% |
| 4 | 831 | 276 | biosynthetic-additional | SDR family NAD(P)-dependent oxidoreductase | 99% |
| 5 | 603 | 200 | regulatory | TetR/AcrR family transcriptional regulator | 99% |
| 6 | 333 | 110 | other | hypothetical protein RER_34830 | 93% |
| 7 | 729 | 242 | other | NUDIX hydrolase | 94% |
| 8 | 1038 | 345 | other | quinolinate synthetase | 100% |
| 9 | 1572 | 523 | biosynthetic-additional | L-aspartate oxidase | 99% |
| 10 | 873 | 290 | other | carboxylating nicotinate-nucleotide diphosphorylase | 99% |
| 11 | 909 | 302 | regulatory | LysR substrate-binding domain-containing protein | 99% |
| 12 | 894 | 297 | other | dihydrodipicolinate synthase family protein | 93% |
| 13 | 1317 | 438 | other | HNH endonuclease signature motif containing protein | 95% |
| 14 | 513 | 170 | other | GNAT family N-acetyltransferase | 94% |
| 15 | 1992 | 663 | other | glycosyltransferase family 39 protein | 99% |
| 16 | 1257 | 418 | other | hypothetical protein | 93% |
| 17 | 504 | 167 | other | GbsR/MarR family transcriptional regulator | 98% |
| 18 | 1032 | 343 | transport | iron-siderophore ABC transporter substrate-binding protein | 99% |
| 19 | 1860 | 619 | other | DUF4012 domain-containing protein | 94% |
| 20 | 16122 | 5373 | biosynthetic | nonribosomal peptide synthetase | 93% |
| 21 | 108 | 35 | other | hypothetical protein N601_21295 | 97% |
| 22 | 555 | 184 | other | DUF5655 domain-containing protein | 91% |
| 23 | 654 | 217 | biosynthetic-additional | GNAT family N-acetyltransferase | 94% |
| 24 | 1320 | 439 | other | MFS transporter | 99% |
| 25 | 1557 | 518 | biosynthetic-additional | pyridoxal phosphate-dependent decarboxylase family protein | 95% |
| 26 | 1332 | 443 | biosynthetic-additional | class I SAM-dependent methyltransferase | 94% |
| 27 | 1320 | 439 | biosynthetic-additional | lipase family protein | 97% |
| 28 | 672 | 223 | other | DUF1345 domain-containing protein | 99% |
| 29 | 630 | 209 | transport | LysE family transporter | 98% |
| 30 | 657 | 218 | biosynthetic-additional | nitroreductase family protein | 99% |
| 31 | 603 | 200 | other | dihydrofolate reductase family protein | 99% |
| 32 | 840 | 279 | other | sulfite exporter TauE/SafE family protein | 98% |
| 33 | 804 | 267 | regulatory | AraC family transcriptional regulator | 97% |
| 34 | 450 | 149 | other | DUF8021 domain-containing protein | 99% |
| 35 | 1290 | 429 | other | glycoside hydrolase family 1 protein | 98% |
| 36 | 3270 | 1089 | biosynthetic-additional | arabinosyltransferase domain-containing protein | 99% |
| 37 | 483 | 160 | other | hypothetical protein | 96% |
| 38 | 1323 | 440 | other | histidinol dehydrogenase | 99% |

Table S3. The structure and mass spectrometry data of NRP analogs.

|  | Fatty acid | A1 | A2 | A3 | A4 | A5 | Theoretical  [M+H]^+^ | Observed Mass  [M+H]^+^ | *Δm*^b)^ (Da) |
| --- | --- | --- | --- | --- | --- | --- | --- | --- | --- |
| ZURJC1 |  | Orn | Thr | Val | Leu | Phe | 593.75 | 593.6 | -0.15 |
| ZURJC2 | Dec | Orn | Thr | Val | Leu | Phe | 748.00 | 747.7 | -0.30 |
| ZURJC3 |  | Orn | Thr | Val | Phe | Val | 579.72 | 579.4 | -0.32 |
| ZURJC4 | Dec | Orn | Thr | Val | Phe | Val | 733.97 | 733.7 | -0.27 |
| ZURJC5 |  | Orn(C)^a)^ | Thr | Val | Leu | Phe(C) | 575.73 | 575.4 | -0.33 |
| ZURJC6 |  | Orn(C) | Thr | Val | Phe | Val(C) | 561.71 | 561.4 | -0.31 |
| ZURJC7 |  | Orn | Thr(C) | Val | Leu | Phe(C) | 575.73 | 575.5 | -0.23 |
| ZURJC8 |  | Orn | Thr(C) | Val | Phe | Val(C) | 561.71 | 561.5 | -0.21 |
| ZURJC9 |  | Lys(C) | Thr | Val | Leu | Phe(C) | 589.75 | 589.6 | -0.15 |
| ZURJC10 |  | Orn(C) | Asn | Val | Leu | Phe(C) | 588.73 | 588.3 | -0.43 |
| ZURJC11 |  | Orn(C) | Thr | Val | Ile | Phe(C) | 575.73 | 575.55 | -0.18 |
| ZURJC12 |  | Orn(C) | Thr | Val | Leu | Trp(C) | 614.76 | 614.5 | -0.26 |
| ZURJC13 |  | Lys(C) | Asn | Val | Ile | Trp(C) | 641.78 | 641.5 | -0.28 |
| ZURJC14 |  | Asp(C) | Thr | Val | Leu | Phe(C) | 576.66 | 576.4 | -0.26 |
| ZURJC15 |  | Asn(C) | Thr | Val | Leu | Phe(C) | 575.70 | 575.4 | -0.30 |
| ZURJC16 |  | Leu(C) | Thr | Val | Leu | Phe(C) | 574.76 | 574.4 | -0.36 |
| ZURJC17 |  | Orn(C) | Thr | Glu | Ile | Phe(C) | 605.72 | 605.3 | -0.42 |
| ZURJC18 |  | Orn(C) | Thr | Val | Glu | Phe(C) | 591.69 | 591.7 | 0.01 |
| ZURJC19 |  | Orn(C) | Thr | Val | Ile | Glu(C) | 557.68 | 557.5 | -0.18 |
| ZURJC20 |  | Orn(C) | Lys | Val | Ile | Phe(C) | 602.81 | 602.8 | -0.01 |
| ZURJC21 |  | Orn(C) | Orn | Val | Ile | Phe(C) | 588.78 | 588.45 | -0.33 |
| ZURJC22 |  | Orn(C) | Arg | Val | Ile | Phe(C) | 630.82 | 630.45 | -0.37 |
| ZURJC23 |  | Orn(C) | His | Val | Ile | Phe(C) | 611.77 | 611.6 | -0.17 |
| ZURJC24 |  | Orn(C) | Thr | Lys | Ile | Phe(C) | 604.78 | 604.4 | -0.38 |
| ZURJC25 |  | Orn(C) | Thr | Orn | Ile | Phe(C) | 590.75 | 590.9 | 0.15 |
| ZURJC26 |  | Orn(C) | Thr | Arg | Ile | Phe(C) | 632.79 | 632.7 | -0.09 |
| ZURJC27 |  | Orn(C) | Thr | His | Ile | Phe(C) | 613.75 | 613.65 | -0.10 |
| ZURJC28 |  | Orn(C) | Thr | Val | Lys | Phe(C) | 590.75 | 590.75 | 0.00 |
| ZURJC29 |  | Orn(C) | Thr | Val | Orn | Phe(C) | 576.73 | 576.75 | 0.02 |
| ZURJC30 |  | Orn(C) | Thr | Val | Arg | Phe(C) | 618.77 | 618.75 | -0.02 |
| ZURJC31 |  | Orn(C) | Thr | Val | His | Phe(C) | 599.72 | 599.6 | -0.12 |
| ZURJC32 |  | Orn(C) | Thr | Val | Ile | Lys(C) | 556.74 | 556.45 | -0.29 |
| ZURJC33 |  | Orn(C) | Thr | Val | Ile | Orn(C) | 542.71 | 542.45 | -0.26 |
| ZURJC34 |  | Orn(C) | Thr | Val | Ile | Arg(C) | 584.75 | 584.7 | -0.05 |
| ZURJC35 |  | Orn(C) | Thr | Val | Ile | His(C) | 565.70 | 565.75 | 0.05 |
| ZURJC36 |  | Orn(C) | Lys | Val | Ile | Lys(C) | 583.80 | 583.45 | -0.35 |
| ZURJC37 | Myr | Orn(C) | Thr | Val | Lys | Phe(C) | 801.14 | 800.8 | -0.34 |

^a)^C: The cyclization site in the backbone.

^b)^*Δm*: measured [M+H]^+^ *m/z* minus theoretical [M+H]^+^ *m/z*.

Table S4. Thermodynamic parameters of ZURJC28 determined by ITC.

| Titrant | Titrand | *Kd*(M)^a)^ | *N*^b)^ | *∆H*(KJ/mol)^c)^ | *∆S*(J/mol·K) |
| --- | --- | --- | --- | --- | --- |
| PG | ZURJC28 | 3.58×10^-7^ | 1.61 | -94.69 | -194.34 |
| PG | ZURJC18 | / | / | / | / |

^a)^*Kd*: Equilibrium dissociation constant, representing the affinity for the target.

^b)^*N*: Molar ratio, indicating the number of titrant molecules bound per titrand molecule.

^c)^*∆H*: Enthalpy, *∆S*: Entropy.
